# Myxomycetes collected in the eastern United States and patterns of relative biodiversity

**DOI:** 10.1080/21501203.2019.1710302

**Published:** 2020-01-05

**Authors:** Steven L. Stephenson, Richard W. Stauffacher, Carlos Rojas

**Affiliations:** a Department of Biological Sciences, University of Arkansas, Fayetteville, AR, USA; b Engineering Research Institute and Department of Biosystems Engineering, University of Costa Rica, San Pedro de Montes de Oca, Costa Rica

**Keywords:** Biodiversity, microbial distribution, Midwest, myxogastrids, Northeast, slime moulds, Southeast, temperate forests

## Abstract

A total of 68 315 digitised records of myxomycetes from the eastern United States were compiled from all readily available sources. After cleaning the database for inconsistencies, 58 594 records remained that were suitable for analysis. A total of 460 different species of myxomycetes were recorded, out of which 410 were classified as rare. Five species were represented by more than 1 500 records and 44 species were represented by only a single record. The states of New York, Virginia and West Virginia have the highest number of records. Almost half of the recognised morphospecies of myxomycetes in the world occur in the eastern United States. A small number of species thrive in different ecological conditions, whereas most species require more specific ecological settings for the formation of fruiting bodies. States associated with high biodiversity have been subjected to more intensive sampling efforts, and southern states seem to have been less studied than northern ones. The project described herein apparently represents one of the few efforts to characterise the myxobiota of a relatively large region of the world.

## Introduction

Myxomycetes have been reported from the eastern United States for more than three centuries, with what was likely to have been *Lycogala* or some other aethalial myxomycete being described in the unpublished notes of John Banister (1654–1692), a young English clergyman in what is now the state of Virginia (Rogers 1981). However, the first extensive records of the group from eastern North America were compiled and published by Lewis David de Schweinitz (1780–1834) in 1822 and 1832. He was followed by a number of other mycologists who collected at least a few myxomycetes along with the higher fungi that represented their primary interest (Martin and Alexopoulos 1969). Among these were Thomas Nuttall (1786–1859), Moses Ashley Curtis (1808–1872), Charles Horton Peck (1833–1917), George Rex (1845–1895), Hugo Bilgram (1847–1932), Roland Thaxter (1858–1932) and Henry Beardslee (1865–1948). These individuals bridged the gap between the early 19th century and the first part of the 20th century, when such individuals as Thomas Huston Macbride (1848–1934), George Willard Martin (1886–1971) and Constantine Alexopoulos (1907–1985) began making major contributions to the study of myxomycetes in the United States.

Many of the specimens collected by the individuals mentioned above and others (both amateurs and professionals) who were familiar enough with myxomycetes to recognise and collect these organisms were deposited in various herbaria. As a result of recent efforts to digitise collection data for fungi and myxomycetes (e.g., the Planetary Biodiversity Inventory project based at the University of Arkansas, the MyCoPortal project based at the University of Illinois and the Global Biodiversity Information Facility [GBIF] project), the records representing these specimens have become more accessible.

Even though this type of data is often spatially biased (Stolar and Nielsen 2014), it is still an important source of information of the distribution of species (Pärtel et al. 2016). In the case of the myxomycetes, there have been few recent attempts to characterise the biota of large geographical areas (e.g. the Neotropics, see Lado and Wrigley de Basanta 2008), but analyses have relied on the literature and may have excluded unpublished data from herbaria. One exception to a more integrated approach was recently carried out in Costa Rica (Lado and Rojas 2018) but the geographical extent of that country limited the potential extrapolation of the analyses. Unfortunately, for most larger geographical and biological units worldwide, there are not enough myxomycete records for meaningful evaluations.

One exception is the eastern section of the United States, where historically, most of the collecting for myxomycetes in North America has been carried out. Comparatively, this area has been studied for much longer and it is biologically much more representative than a small tropical country. Also, the known influence of seasonality on biodiversity patterns (Tonkin et al. 2017) along with the high productivity of seasonal temperate forests during the growing season and its impact on biodiversity (Gillman et al. 2014), make this geographical area very interesting from a biological point of view. In this manner, considering that there is a robust dataset on myxomycetes for this region, the objectives of the project reported herein were first to determine what species of myxomycetes have been recorded from the eastern United States, based on specimens that have been digitised in recognised herbaria, and second to perform a preliminary analysis of the record distribution and explore biologically meaningful patterns.

## Materials and methods

**Figure 1.**
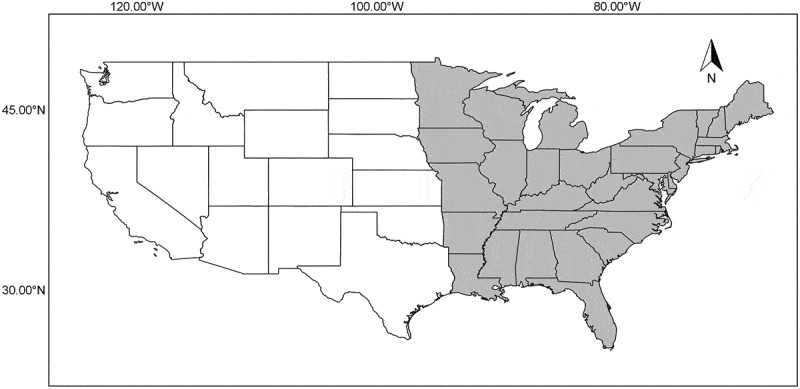
Map of the continental section of the United States showing the eastern area (highlighted in grey) as defined in the present study

All records from the states of Alabama, Arkansas, Delaware, Florida, Georgia, Illinois, Indiana, Iowa, Kentucky, Louisiana, Maine, Maryland, Massachusetts, Michigan, Minnesota, Mississippi, Missouri, New Hampshire, New Jersey, New York, North Carolina, Ohio, Pennsylvania, Rhode Island, South Carolina, Tennessee, Vermont, Virginia, West Virginia and Wisconsin as well as the District of Columbia were assembled into a single database. These states and the District of Columbia were considered to represent the eastern United States (Figure 1). A total of 68 315 records were obtained from three sources mentioned in the introductory section. The actual herbaria providing the largest numbers of records were those of the University of Arkansas (UARK), the National Fungus Collections (BPI), Harvard University (FH), the New York Botanical Garden (NY), the University of Michigan (MICH) and the Academy of Natural Sciences (PH). However, at least a few records were obtained from almost 100 different herbaria.

The initial database included numerous records identified only to genus, invalid or doubtful names that had been applied to particular specimens, synonyms of currently accepted names and duplicate records that appeared in more than one of the sets of data compiled from the various sources. After these records had been removed from the initial database, a total of 58 594 records remained that were suitable for analysis. The nomenclatural standard used for taxonomic names in the project followed Lado (2005–2019) with two exceptions. These were *Perichaena liceoides* Rostaf. and *Stemonitis nigrescens* Rex. Lado includes the former in *P. corticalis* (Batsch) Rostaf., and he does not consider the latter to be distinct from *Stemonitis fusca* Roth. *P. liceoides* and/or *S. nigrescens* have been regarded as distinct species in a number of other publications on the myxomycetes (e.g., Martin and Alexopoulos 1969; Ing 1999; Poulain et al. 2011), and that was the approach used herein. Different identifications of equivalent material made by different researchers cannot be ruled out and this could be a potential source of inconsistencies. However, it is beyond the scope of this study to deal with such shortcoming.

An analysis of diversity patterns was carried out with the complete dataset using the programs SpadeR and iNEXT (Chao et al. 2015; Hsieh et al. 2016). Based on a simplified version of the ACOR scale provided by Stephenson et al. (1993), the distribution of records was divided in two groups (abundant and rare) and the coverage shown by the data within each group was calculated. For this, the complete dataset was divided using the 1.5% threshold normally used between the “common” and “occasional” categories to create extended versions of the “abundant” and “rare” categories. With this approach, it was possible to have an idea of the potential number of species not yet recorded in the dataset based on the structure of the partial sub datasets. Also, the expected species richness was calculated using the Chao 1 bias-corrected indicator and the percentual completeness of the dataset was estimated. Even though the dataset is highly heterogeneous, such an estimate could provide a figure for future comparisons. A rarefaction curve was constructed using the relationship between the number of records and the accumulated number of species to observe the potential contribution of doubling the collecting effort. Also, a Hill number-based diversity profile was created to compare the studied dataset with published results from Costa Rica (Lado and Rojas 2018). This type of profile is useful to visually assess some characteristics of a biological community such as species richness and evenness/dominance in one figure (the axis y is multiscale, but it is referred to as “diversity”) and can also be used to display survey completeness at the different q values. Even though the spatial scale of that comparison is very different, Costa Rica is perhaps the only other region where the myxomycetes have been studied enough to allow a simple comparison to be meaningful.

Finally, results in the original database were arranged by state and a series of ecological and effort estimators were calculated. The latter included species richness, the ratio of the number of records and the number of species (herein referred to as taxonomic diversity index or TDI), the Simpson’s and Shannon’s Diversity Indices, Shannon’s Evenness Index (Jʹ) and the maximum number of species to be expected using the Chao 1 estimator. Even though the latter should be interpreted carefully with such a heterogeneous dataset, it was included to provide a figure for future comparative purposes. Both of the Shannon´s estimators were used to create maps in QGIS, v. 3.8 (www.qgis.org) for graphical interpretation of both effort and distribution of records. Finally, a comparison of effort and diversity profiles associated between the state of West Virginia and Costa Rica was carried out. The former has been well studied for myxomycetes and it is about the same size of Costa Rica, a tropical well studied country as well. This comparison simply aimed at establishing similarities/differences between the two without the problems of scale.

## Results

**Figure 2.**
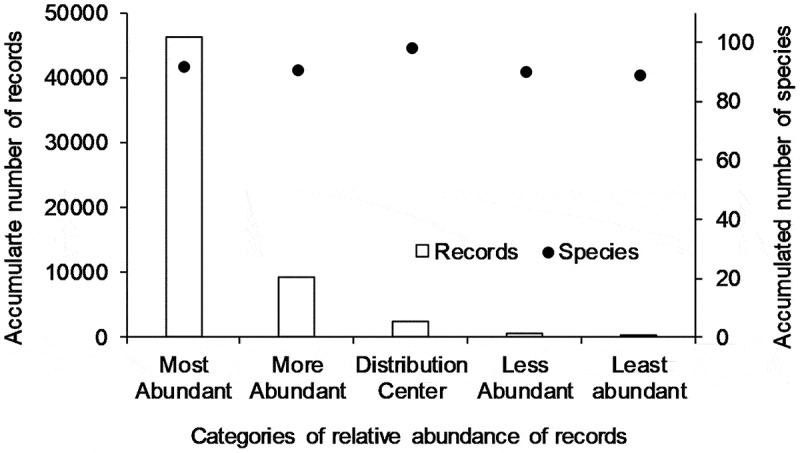
Pattern of decreasing number of accumulated records of myxomycetes associated with lower categories of relative abundance observed in the studied dataset, despite the similar number of species included in each category

The 58 594 records in the final database represented a total of 460 different species. Five species were represented by more than 1 500 records. These were *Arcyria denudata* (L.) Wettst., *Lycogala epidendrum* (L.) Fr., *Arcyria cinerea* (Bull.) Pers., *Metatrichia vesparia* (Batsch) Nann.-Bremek. ex G.W. Martin & Alexop. and *Trichia favoginea* (Batsch) Pers. In contrast, 44 species were represented by only a single record. The overall distribution of species with respect to the number of records is presented in Figure 2. The majority of the specimens in both the initial database and the final database had been collected since 1900, but an appreciable number of specimens (3 920) were collected prior to the beginning of the twentieth century. The oldest specimens considered in the present study were from 1815, with the period of 1815 to 1849 represented by 26 specimens and the period of 1850–1875 represented by 72 specimens. As such, the period between 1875 and 1900 was represented by the largest number of pre-twentieth century specimens. Interestingly, the most prolific pre-twentieth century collectors, based on specimens in the final database, were George Rex (425 specimens), Roland Thaxter (389 specimens), Hugh Bilgram (328 specimens) and Henry Beardslee (236 specimens). Although these numbers do not reflect the total number of specimens they collected, since not all of their specimens would be in the records used to compile the database, they do give some indication of their level of activity.

**Figure 3.**
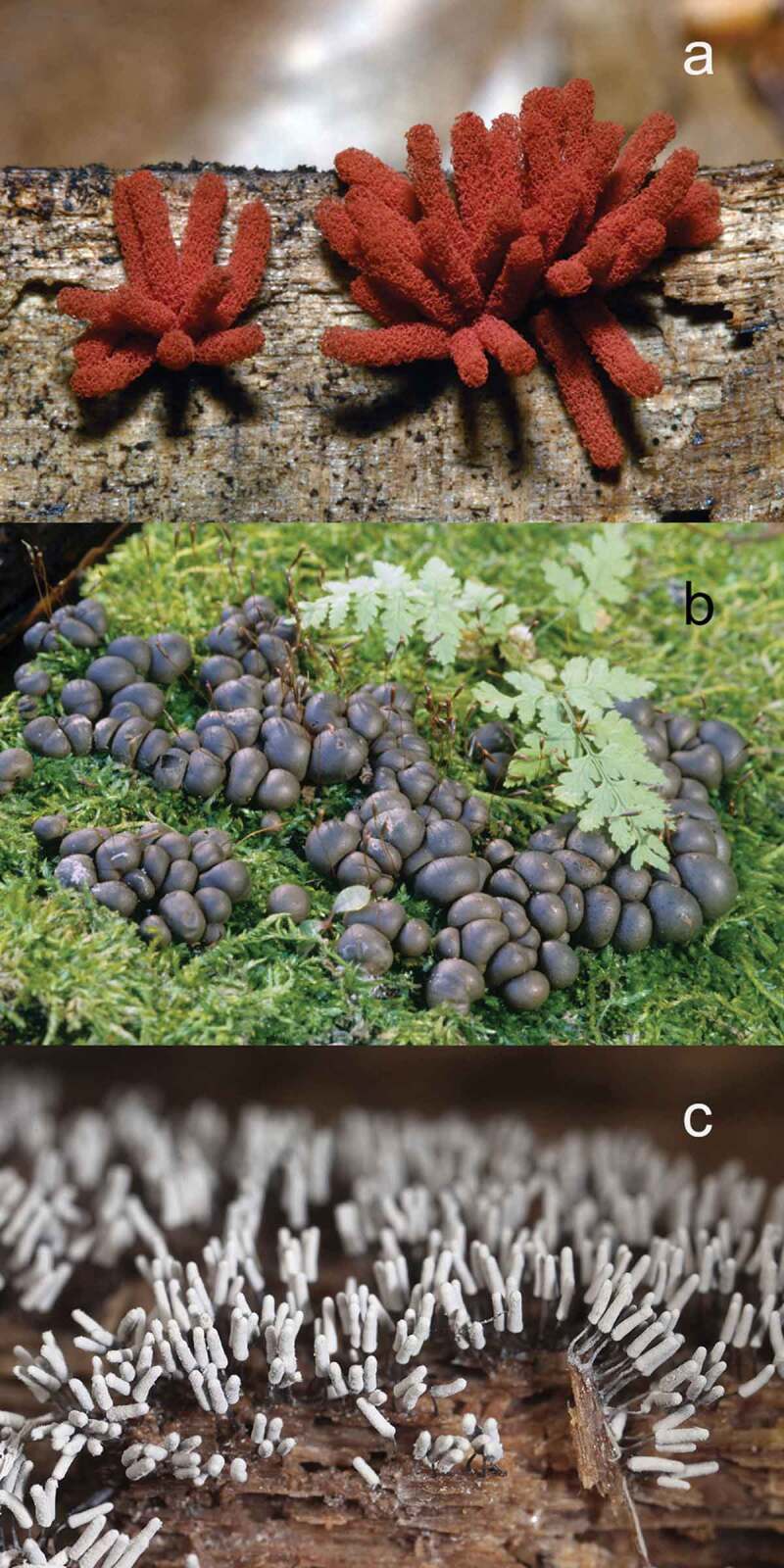
The three species of myxomycetes considered to be abundant in the analysis carried out during the present study. a) *Arcyria denudata*, b) *Lycogala epidendrum* and c) *Arcyria cinerea.*

When the simplified version of the ACOR scale was applied to the final database of 58 594 records, 11 species were found to be abundant and 449 were rare. In the former group, three species (*Arcyria denudata, Lycogala epidendrum* and *Arcyria cinerea* [Figure 3]) were very abundant, and eight species (including *Hemitrichia calyculata* (Speg.) M.L. Farr, *Fuligo septica* (L.) F.H. Wigg., *Stemonitis fusca* and *Physarum viride* (Bull.) Pers.) were less abundant (~common). In the rare group, 39 species (including *Physarum leucophaeum* Fr. & Palmquist, *Clastoderma debaryanum* A. Blytt and *Cribraria argillacea* (Pers. ex J.F. Gmel.) Pers.) would be considered less rare (~occasional, see Appendix), and the remaining 410 species would be considered as very rare.

**Figure 4.**
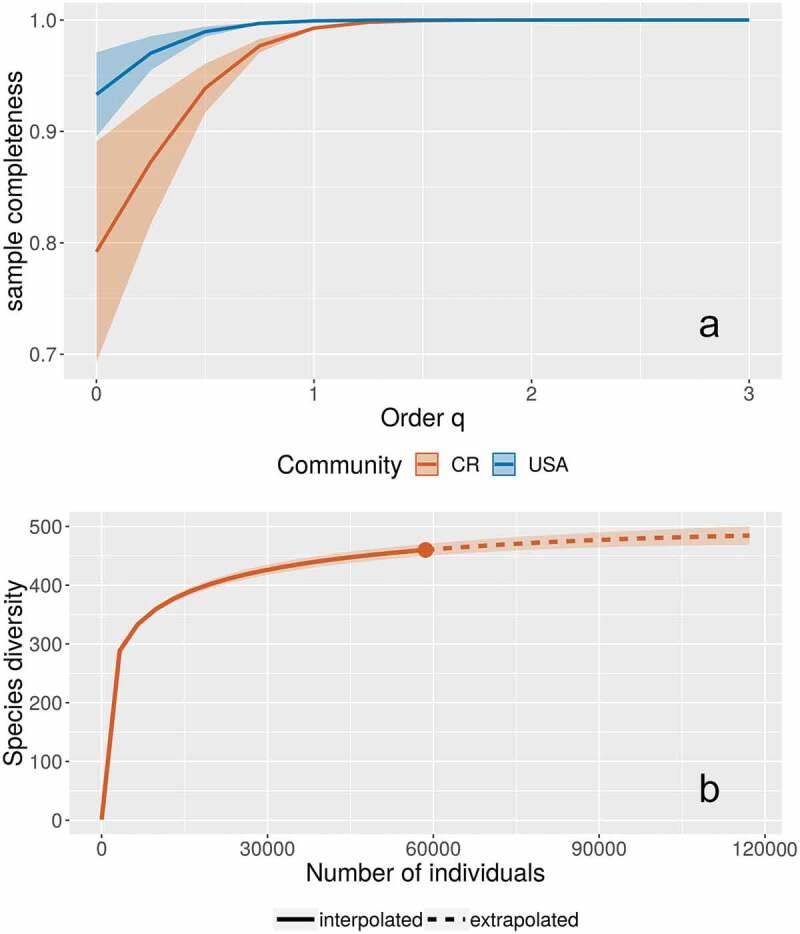
Survey completeness at different Hill numbers of order q for the myxomycete datasets of Eastern USA (abbreviated as USA) and Costa Rica (CR, a) and rarefaction curve for the first one, showing an extrapolation based on doubling the dataset (b). Shading corresponds with the 95% confidence intervals

According to the coverage analyses based on the Chao 1 bias-corrected estimator, 100% of the abundant species and about 93% of the rare species have been recorded in the eastern part of the United States. A value of 490 species was calculated as the maximum for the complete dataset (with a 95% confidence interval between 475–525 species), indicating that, with the effort and techniques used to generate the studied dataset, approximately 94% of the species have already been noted. These results were observed in Figure 4(a), where the survey was incomplete only at the order q value of 0, associated with species richness. Order q values of 1 and 2, indicating species with “typical” abundances and effective number of dominant species, respectively, reached the highest completeness levels. Interestingly, for the Costa Rican dataset used for comparison, incompleteness was observed at both the order q value of 0 and 1. The rarefaction curve constructed with the dataset from the eastern part of the United States (Figure 4(b)) showed that the accumulation of species started levelling at around 15 000 records, and that doubling the effort would account, albeit slowly, for the species not yet recorded.

**Figure 5.**
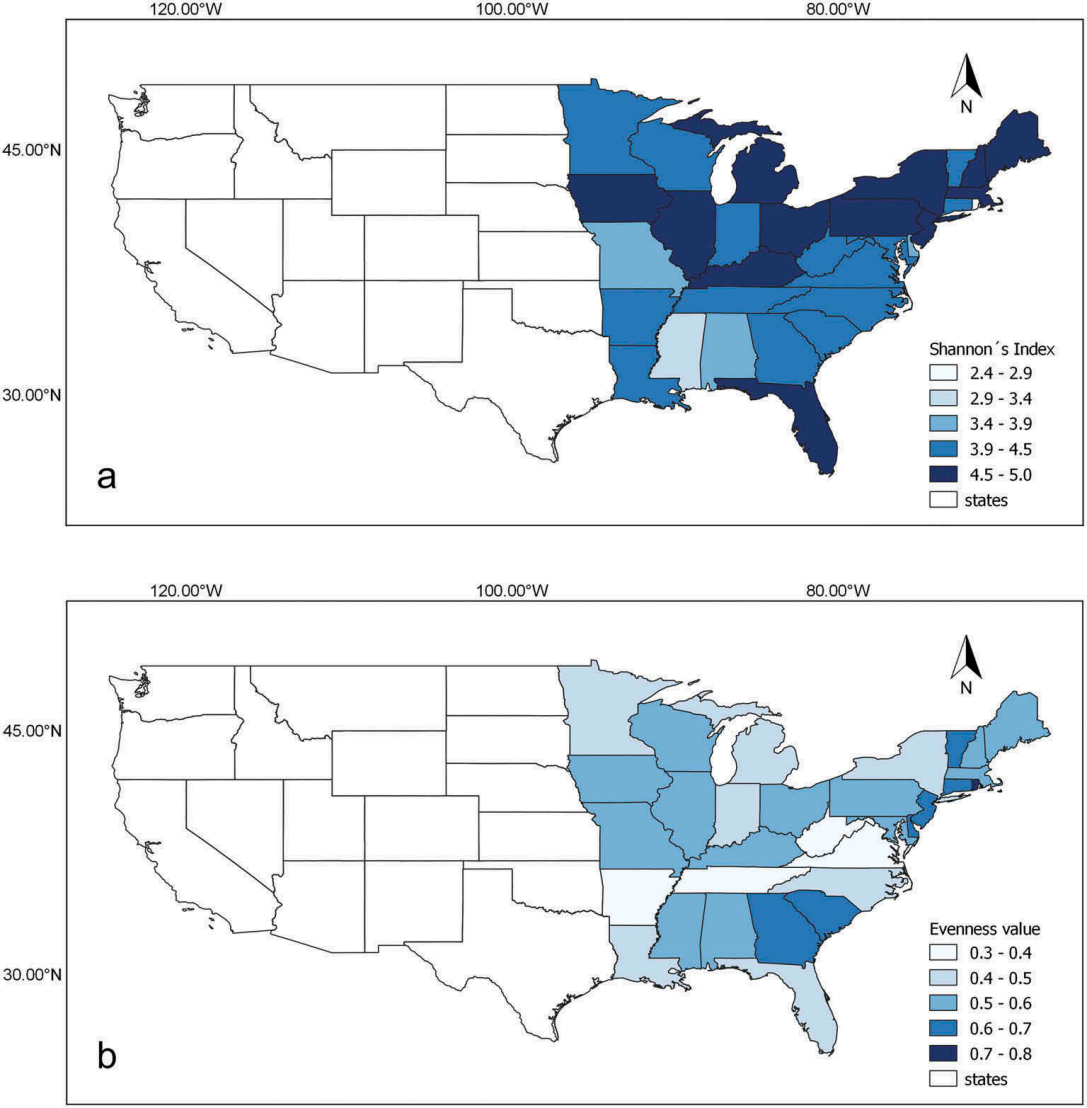
Maps of the continental United States highlighting the eastern part of the country studied in the present study in terms of the Shannon´s Index (a) and Evenness (b) shown by the myxomycete data arranged by states

The five states with the most records in the database were New York, Virginia, West Virginia, Pennsylvania and Arkansas (Table 1). For these states, the number of species recorded ranged between 178–247 and the number of records per species ranged between 21–30. Interestingly, for a second group of states including Iowa, North Carolina and Tennessee, the number of recorded species was higher than 178 despite a lower number of total records of myxomycetes. Both evaluated Indices of Diversity showed the highest value in Iowa and the lowest in Rhode Island, where the effort was also the lowest (Figure 5(a)). Evenness showed the lowest value in Arkansas and the highest in Rhode Island (Figure 5(b)). Using the Chao 1 value as the maximum number of species, the state with the highest survey completeness was Vermont (99%) and the one with the lowest was Mississippi (45%).

**Table 1.** Summary of ecological indicators associated with the myxomycete distribution observed in each state evaluated during the present study. TDI = taxonomic diversity index

State	Ecological indicators						
	Number of Records	Species Richness	TDI (records/species)	Simpson’s Diversity Index	Shannon’s Diversity Index	Shannon’s Evenness Index (Jʹ)	Chao 1 Value
NY	7274	247	29.4	0.988	4.793	0.493	272
VA	6346	223	28.5	0.976	4.335	0.345	234
WV	5214	215	24.3	0.978	4.372	0.374	219
PA	4608	222	20.8	0.989	4.858	0.585	235
AR	3742	178	21.0	0.963	3.961	0.298	197
IA	3393	235	14.4	0.991	4.97	0.618	262
NC	3169	196	16.2	0.979	4.402	0.421	202
TN	2896	195	14.9	0.974	4.354	0.403	219
IL	2894	176	16.4	0.985	4.594	0.569	190
MI	2882	198	14.6	0.983	4.507	0.463	213
MA	2296	170	13.5	0.985	4.499	0.532	177
FL	2068	180	11.5	0.984	4.504	0.508	201
OH	1949	179	10.9	0.983	4.526	0.522	197
NH	1582	141	11.2	0.985	4.466	0.622	148
ME	1495	165	9.1	0.984	4.518	0.563	181
LA	1214	148	8.2	0.978	4.295	0.502	157
IN	1098	137	8.0	0.973	4.182	0.485	158
MN	1054	119	8.9	0.974	4.068	0.495	131
NJ	1048	158	6.6	0.986	4.609	0.644	170
MD	1010	145	7.0	0.982	4.41	0.572	165
KY	855	145	5.9	0.983	4.458	0.604	154
GA	719	137	5.2	0.983	4.444	0.626	155
VT	707	96	7.4	0.979	4.127	0.653	96
WI	635	101	6.3	0.976	4.066	0.583	129
MO	329	88	3.7	0.965	3.926	0.590	103
CT	315	71	4.4	0.975	3.936	0.721	77
SC	259	76	3.4	0.975	3.979	0.704	87
AL	250	59	4.2	0.951	3.504	0.573	68
DE	136	51	2.7	0.965	3.617	0.730	69
MS	116	45	2.6	0.944	3.306	0.620	98
DC	69	35	2.0	0.949	3.304	0.778	50
RI	27	13	2.1	0.894	2.394	0.843	17

**Figure 6.**
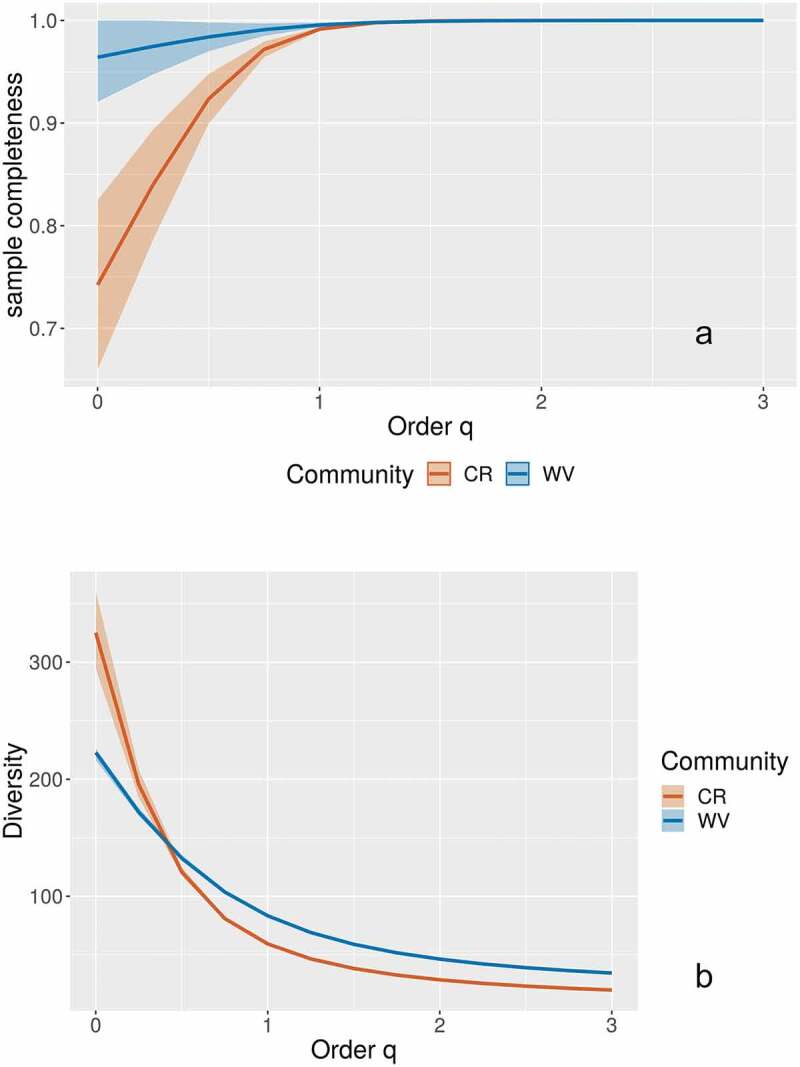
Survey completeness at different Hill numbers of order q for the myxomycete datasets of West Virginia (abbreviated as WV) and Costa Rica (CR, a) and diversity profiles for the same datasets (b). Shading corresponds with the 95% confidence intervals

Finally, when the subdataset associated with West Virginia was compared with a similar one from Costa Rica, results showed that the completeness of the surveys using order q values was higher at each level for West Virginia than for Costa Rica (Figure 6(a)). Even though the species diversity was higher in Costa Rica, the West Virginia dataset showed higher values of “typical” and dominant species as observed in the curve at order q values of 1 and 2, respectively (Figure 5(b)). In this case, the Shannon´s Index of diversity/evenness values were 4.4/0.38 for West Virginia and 4.0/0.25 for Costa Rica.

## Discussion

Specimens of myxomycetes that develop under natural conditions in the field can be collected in the same way as the fruiting bodies of fungi, and this was certainly the case for specimens collected during the nineteenth century. However, after Gilbert and Martin (1933) introduced what became known as the moist chamber culture technique, field-collected specimens could be supplemented by specimens appearing in laboratory cultures prepared with samples of bark, dead leaves and various type of plant debris. During the second half of the twentieth century and continuing on into the twenty-first century, an increasing number of records in herbaria were also certainly represented by specimens obtained from moist chamber cultures. In fact, most records now known for species in such genera as *Echinostelium, Licea* and *Macbrideola* are from moist chamber cultures. Such a methodological bias has likely influenced the relative abundance of these genera in most myxomycete surveys. Even though some very small fruiting bodies can be observed in the field, the probability to find them in natural conditions is lower than the probability to find the bigger, brighter forms.

In the final database compiled in the present study, *Echinostelium* (520 specimens), *Licea* (796 specimens) and *Macbrideol*a (82 specimens) represented about 2.4% (a total of 1 398 specimens) of the total number. Not all of these were obtained from moist chamber cultures, since some of the larger species of *Licea* (e.g., *Licea minima*) are easily detected on pieces of substrate material (bark in this case) brought back to the laboratory and examined under a stereomicroscope because of the presence of another species observed directly in the field. This is certainly the case for other species that produce very small fruiting bodies. One example is *Barbeyella minutissima* Meyl., which was represented by 68 specimens in the final database. There are only a couple of records of this species appearing in moist chamber cultures, so it is likely that virtually all (if indeed not all) of the 68 specimens had developed in the field under natural conditions. In this manner, the elusiveness of most small myxomycetes along with the use of traditional techniques has played a role in the accumulation of ecological and biogeographical information on myxomycetes.

The 460 species recorded for the eastern section of the United States represent 42.4% of the 1 084 morphological species of myxomycetes currently (as of early 2019) known worldwide (nomen.eumycetozoa.com; Lado 2005–2019). According to the results presented herein with about 490 species potentially expected in the studied area, it is possible that about at least 30 more species could be found in it (and perhaps even more based on the confidence intervals). This number is a simple estimate that depends heavily on the homogeneity of the surveys and given the fact that records analysed herein come from very different experimental designs, such values should be carefully interpreted. Despite such shortcoming, results showed that the eastern section of the United States has been more thoroughly studied than a small and easier to study country like Costa Rica. In that sense, there is little doubt that more than 460 species have been recorded from the eastern states, since not all species (especially those that are exceedingly rare) would have been represented by specimens in the herbaria providing the data compiled in the present study or simply identified differently by researchers. Moreover, it is very likely that even more than 490 species of myxomycetes could be recorded in this area if a combination of techniques, along with standard efforts focused on undersampled microhabitats could be carried (see Wrigley de Basanta and Estrada-Torres 2017). In this manner, the value of 30 more expected species is simply a reference based on the normal recording techniques and morphological approach used by classical researchers.

The number of species known for the entire United States must be dramatically higher, since there are some myxomycetes associated with special habitats which do not occur or occur only rarely in the eastern half of the country. The most important of these are the snowbank habitats which occur in alpine areas of the mountains of the western United States. Although a few species of nivicolous myxomycetes (e.g., *Lamproderma ovoideum* Meyl., *Lepidoderma carestianum* (Rabenh.) Rostaf., *Prototrichia metallica* (Berk.) Massee and *Trichia alpina* (R.E. Fr.) Meyl.) are known from the eastern United States, albeit from a very limited number of records, there are an appreciable number of other species not known from the eastern United States. The same situation applies to those myxomycetes associated with the desert habitats of the southwestern United States. Several species (e.g., *Didymium eremophilum* M. Blackw. & Gilb. and *Didymium mexicanum* G. Moreno, Lizárraga & Illana) have been recorded from these deserts and appear to be restricted to such habitats (Novozhilov et al. 2003).

There is little doubt that those species known to produce large and/or colourful fruitings (either individual fruiting bodies in the case of such examples as *Lycogala epidendrum* and *Fuligo septica*) or extensive groups of fruiting bodies (as is the case for *Stemonitis fusca* and *Physarum viride*) are greatly overrepresented in the final database. However, these same data also suggest that many species of myxomycetes are indeed rarely collected, in part because of the small size of their fruiting bodies but also due to the fact that they are not particularly common. It is perhaps noteworthy that *Ceratiomyxa fruticulosa* (O.F. Müll.) T. Macbr. falls into the less rare category. In the experience of the first author, who has collected and studied myxomycetes in the eastern United States for more than 40 years, this species is often exceedingly abundant. However, it doesn’t produce what might be considered “attractive” fruiting bodies, and if not handled properly, fruitings don’t preserve as well as those of most myxomycetes. On the basis of molecular studies, members of the genus *Ceratiomyxa* are now known to be a sister group to the other “true” myxomycetes, but traditionally it has been considered to be a myxomycete and thus subject to being collected in the same manner.

Nevertheless, the information presented herein does provide an overview of the assemblage of myxomycetes associated with habitats in the eastern United States. However, the larger numbers also would be expected to reflect relative abundance. It is noteworthy that a high level of rare species (about 89%) was observed for the eastern United States. When a well surveyed state such as New York is analysed, the number of rare species is lower (about 79% and 99% of rare species recorded, not shown before) indicating that spatial heterogeneity of habitats, and the respective effort to study them, may play a role in the process of recording myxomycete species. In the present case, the species represented by the very largest numbers in the database are common to abundant in nature. Evidence of this is the fact that they are almost invariably present at any locality surveyed for myxomycetes. It is interesting, however, to observe that the largest sampling efforts and highest values of the Shannon´s Diversity Index were associated with northern states and that some other areas such as the states of Alabama, Mississippi and Missouri, have been understudied. Also, despite the good sampling effort, it is interesting to observe that myxomycete assemblages in Arkansas, Tennessee, Virginia and West Virginia showed low ecological evenness, suggesting that sampling has been conducted in few locations that reflect a low number of ecological situations. The comparison of patterns between West Virginia and Costa Rica seemed to point in the same direction, since myxomycetes in the latter have been studied in many more locations than in the former.

The database compiled in the present study has the potential for serving as the basis for comparative studies of the assemblages of myxomycetes associated with other regions of the world where these organisms are sufficiently well known. The first two examples that come to mind are Western Europe and Eastern Asia, where the climatic conditions and vegetation are somewhat similar to those of the eastern United States. However, comparisons with the assemblages associated with very different climatic conditions and vegetation (e.g., the preliminary comparison made with Costa Rica considered herein) also would be worthwhile, since they would allow a more complete understanding of the global patterns of distribution and biodiversity of one group of microorganisms – the myxomycetes.

## Appendix

List of myxomycete species in the less abundant and less rare categories (equivalent to common and occasional in the ACOR scale) recorded in the eastern United States, arranged by frequency.

Less abundant species (~common)

Metatrichia vesparia, Trichia favoginea, Hemitrichia calyculata, Fuligo septica, Stemonitis fusca, Physarum viride, Stemonitis axifera, Hemitrichia clavata

Less rare species (~occasional)

Hemitrichia serpula,Trichia varia,Tubifera ferruginosa, Ceratiomyxa fruticulosa, Arcyria incarnata, Cribraria cancellata, Physarum album, Diderma effusum, Arcyria obvelata, Physarum bivalve, Physarum cinereum, Stemonitopsis typhina, Stemonitis splendens, Cribraria intricata, Trichia decipiens, Leocarpus fragilis, Physarum globuliferum, Trichia scabra, Diderma testaceum, Diachea leucopodia, Didymium squamulosum, Arcyria pomiformis, Collaria arcyrionema, Didymium melanospermum, Diderma spumarioides, Perichaena chrysosperma, Mucilago crustacea, Reticularia splendens, Echinostelium minutum, Didymium iridis, Physarum polycephalum, Comatricha nigra, Craterium leucocephalum, Arcyria stipata, Trichia contorta, Oligonema flavidum, Physarella oblonga, Craterium obovatum, Perichaena depressa
